# Adipocyte integrin-linked kinase plays a key role in the development of diet-induced adipose insulin resistance in male mice

**DOI:** 10.1016/j.molmet.2021.101197

**Published:** 2021-02-26

**Authors:** Aimée R. Bugler-Lamb, Annie Hasib, Xiong Weng, Chandani K. Hennayake, Chenshi Lin, Rory J. McCrimmon, Roland H. Stimson, Michael L.J. Ashford, David H. Wasserman, Li Kang

**Affiliations:** 1 Division of Systems Medicine, School of Medicine, University of Dundee, Dundee, Scotland, UK; 2 Center for Cardiovascular Science, University of Edinburgh, Edinburgh, Scotland, UK; 3 Department of Molecular Physiology and Biophysics and Mouse Metabolic Phenotyping Center, Vanderbilt University, Nashville, TN, USA

**Keywords:** Adipose tissue, Extracellular matrix, Insulin clamp, Insulin resistance, Integrin-linked kinase

## Abstract

**Objective:**

Increased deposition of the extracellular matrix (ECM) in adipose tissue (AT) during obesity contributes to insulin resistance. The integrin receptors transmit changes in the extracellular environment causing corresponding intracellular adaptations. Integrin-linked kinase (ILK), an adaptor protein, is a central hub for intracellular signaling of integrins. This study determined the role of ILK in adipose function and insulin resistance.

**Methods:**

The pathogenic role of ILK in obesity and insulin resistance was studied in human adipose tissue and adipocyte-specific ILK-deficient mice (ILK^lox/lox^*AdCre*). ILK^lox/lox^*AdCre* mice together with wild-type littermates (ILK^lox/lox^) were fed a chow diet or 60% high-fat (HF) diet for 16 weeks. In vivo insulin sensitivity was determined by hyperinsulinemic-euglycemic clamps.

**Results:**

AT ILK expression was increased by HF diet feeding in mice and increased in visceral fat of morbidly obese humans. The HF-fed ILK^lox/lox^*AdCre* mice displayed reduced fat mass and improved glucose tolerance relative to the HF-fed ILK^lox/lox^ mice. During a hyperinsulinemic-euglycemic clamp, the HF-fed ILK^lox/lox^*AdCre* mice exhibited partially improved insulin resistance in AT. Lipolysis was suppressed to a greater extent by insulin and glucose uptake in brown AT (BAT) increased. Increased inhibition of lipolysis may have been attributed to increased vascularization in white AT, while increased glucose uptake in BAT was associated with increased Akt phosphorylation and P38/JNK dephosphorylation. Notably, AT insulin sensitivity in lean mice was not affected by ILK deletion. Moreover, reduced fat mass in the HF-fed ILK^lox/lox^*AdCre* mice may have been attributed to decreased free fatty acid uptake into adipocytes via the downregulation of CD36 gene expression. Consistent with the results in the mice, knockdown and knockout of ILK in 3T3-L1 cells decreased lipid accumulation and CD36 gene expression during adipogenesis.

**Conclusions:**

These data show that adipocyte ILK is important for regulating HF diet-mediated insulin resistance in AT in a manner consistent with AT function.

## Highlights

• ILK protein increased in visceral adipose tissue of obese humans and mice.
• Mice lacking adipocyte ILK had less fat and improved glucose tolerance in obesity.
• Adipocyte ILK deletion improved anti-lipolytic action of insulin in obese mice.
• Adipocyte ILK deletion stimulated brown adipose tissue glucose uptake in obese mice.

## Abbreviations

AT: adipose tissue
AUC: area under the curve
BAT: brown adipose tissue
CLAMS: comprehensive lab animal monitoring system
2[^14^C]DG: 2-[^14^C]deoxyglucose
ECM: extracellular matrix
EndoRa: endogenous glucose production rate
HF: high fat
ICv: hyperinsulinemic-euglycemic clamp
ILK: integrin-linked kinase
IPGTT: intraperitoneal glucose tolerance test
NEFA: non-esterified fatty acid
Ra: glucose appearance rate
Rd: glucose disappearance rate
Rg: metabolic index for tissue glucose uptake
RER: respiratory exchange ratio
RBC: red blood cells
WAT: white adipose tissue

## 1 Introduction

Obesity is known to be a risk factor for developing insulin resistance, a hallmark of type 2 diabetes [1,2]. In adipose tissue, obesity-associated insulin resistance correlates with increased deposition of collagens within the extracellular matrix (ECM) [3]. Of note, obese insulin-resistant subjects display increased collagen deposition in their adipose tissue compared to obese insulin-sensitive subjects with a similar body mass index [4], suggesting that adipose tissue ECM remodeling plays an important role in the pathogenesis of insulin resistance independent of obesity. It is currently unknown, however, how remodeling the ECM within adipose tissue affects the pathophysiology of obesity and insulin resistance.

The ECM is important for maintaining a suitable environment for proliferation, survival, migration, and differentiation of cells by regulating growth hormones, receptors, and pH outside the cells [5]. Through substrate-adhesion molecules, the ECM regulates intracellular signaling [6,7], with the main substrate adhesion molecules being integrins [8]. Integrins are a family of >20 heterodimers that makes interpreting the deletion of any one subunit difficult. One of the main adaptor proteins that link integrins to intracellular signaling is integrin-linked kinase (ILK), which together with PINCH and Parvin forms the IPP complex [9] and binds to the β subunit of integrins [10,11]. ILK is highly conserved and widely expressed throughout the body and carries out multiple functions, one of which is the organization of the actin cytoskeleton. It has been shown that whole-body ILK-deficient mice die at the embryotic, peri-implantation stage indicating a vital role of ILK in development [12]. However, recent studies using conditional ILK knockout mice suggest genetic deletion in the kidneys prevents angiotensin II-induced renal inflammation [13]. Moreover, tissue-specific deletion of ILK in skeletal muscle or liver ameliorated high-fat (HF) diet-induced insulin resistance in C57BL/6 mice in the respective tissues [14,15].

Adipose tissue is an endocrine organ that plays important roles in regulating inflammation and metabolism. In addition to their role in fat storage [16], adipocytes express and secrete adipokines, cytokines, and hormones such as leptin and adiponectin, which affect appetite, thermogenesis, glucose metabolism, fatty acid oxidation, and insulin sensitivity [17,18]. Despite the central role of ILK in the adaptation to a changing extracellular environment, its role in adipose tissue during the challenge of obesity is unknown. Using adipocyte-specific ILK-deficient mice, we aimed to understand the role of ILK in adipose tissue in diet-induced obesity and insulin resistance.

## 2 Materials and methods

### 2.1 Human tissue sample collection and preparation

Paired human subcutaneous and visceral adipose tissue samples were obtained from subjects undergoing elective abdominal surgery at the Royal Infirmary of Edinburgh. Local ethical approval was obtained and informed consent was obtained from each subject. The samples were immediately frozen at −80 °C until analysis. Tissue samples were lysed using lysis buffer supplemented with 92 mg/ml of sucrose, 0.1% beta-mercaptoethanol, 1 mM of sodium vanadate, 1 mM of benzamidine, 0.1 M of PMSF at a pH of 7.4. The tissues were then homogenized and the protein concentration was measured using Bradford reagent (#B6916; Sigma).

### 2.2 Mouse model

The generation of ILK-floxed mice was previously described in detail [12], where the ILK gene was flanked by loxP sequences on both sides of exon 2. To generate C57BL/6 adipocyte-specific ILK-deficient mice (ILK^lox/lox^*AdCre*), the ILK-floxed mice were crossed with transgenic mice expressing *Cre* recombinase under the control of adiponectin promoter (#010803; Jackson Laboratory). The ILK^lox/lox^*AdCre* mice were confirmed by genotyping for positive adiponectin *Cre* and homozygous for ILK-floxed genes. The control mice used in these experiments were littermates without the expression of adiponectin *Cre* (ILK^lox/lox^). The mice were fed either a chow diet (D/811004; DBM) or a 60% high-fat (HF) diet (824054; SDS) for 16 weeks starting at 6 weeks of age. All of the mice were housed in a temperature (22 ± 1 °C) and humidity-controlled room with a 12-h light/dark cycle. The mice had free access to food and water. Only male mice were used in this study due to their high susceptibility to a robust insulin-resistant phenotype after HF feeding. All the animal procedures complied with the ARRIVE guidelines and were approved by the UK Home Office and the Animal Care and Use committee of the University of Dundee.

### 2.3 Comprehensive lab animal monitoring system (CLAMS)

The mice at 20 weeks of age were housed in a CLAMS (Columbus Instruments) under ambient temperatures (22 ± 1 °C) for 72 h to monitor their food intake, water intake, respiratory exchange ratio (RER), energy expenditure, and ambulatory activity. Light cycle, room temperature, and access to food and water were maintained. Body weight and composition were measured before and after the experiment.

### 2.4 Hyperinsulinemic-euglycemic clamp

Five days prior to the hyperinsulinemic-euglycemic clamp (ICv), the mice underwent surgery for vascular cannulations in both the jugular vein (for infusing glucose, insulin, and red blood cells (RBC)) and the carotid artery (for blood sampling) [19]. The mice were fasted for 5 h prior to ICv, and [3-^3^H]glucose was infused to measure glucose flux rates [14]. Insulin was infused at 4 mU/kg/min throughout the ICv. The blood glucose was clamped at 6 mmol/L using a glucose infusion rate that varied in accordance with the arterial blood glucose levels measured every 10 min. Washed RBCs were infused at a constant rate, which was calculated to equal the RBCs removed during the ICv. Blood samples were taken every 10 min between 80 and 120 min to assess glucose flux rates. At 120 min, 481 kBq of 2-[^14^C]deoxyglucose (2[^14^C]DG) was administered as a bolus through the jugular vein to measure the glucose uptake in individual tissues. Blood samples were taken between 120-155 min to determine the radioactivity of 2[^14^C]DG after which the mice were sacrificed and their tissues excised.

### 2.5 ICv plasma and tissue sample processing and glucose flux rate determination

The radioactivity of [3-^3^H]glucose and [^14^C]2DG was measured in plasma and [^14^C]2DG-phosphate was measured in the tissue samples by liquid scintillation counting as previously described [20]. Whole-body glucose appearance (Ra) and disappearance (Rd) were determined using non-steady-state equations for isotope dilution [21]. Endogenous glucose appearance (EndoRa) was determined by subtracting the glucose infusion rate from Ra. The metabolic index for tissue glucose uptake (Rg) was determined as previously described [22].

Non-steady-state calculation of glucose flux

$$Ra=\frac{I-Vd.A.\frac{dSA}{dT}}{SA}\;Rd=Ra-Vd.\frac{dA}{dT}$$

*Ra* and *Rd*: glucose appearance and disappearance rates (mg/kg/min), *I*: tracer infusion rate (dpm/min), *Vd*: volume distribution of glucose, *A*: concentration of glucose (mg/dL), *SA*: specific activity of glucose (dpm/mg), and *T*: time (min).

Tissue-specific glucose uptake (Rg) calculation

Rg=[C14]2DGPintissueAUCplasma[C14]2DG.averageglucoseconcentration

*[*^*14*^*C]-2DGP*: ^14^C-2-deoxyglucose phosphate; *[*^*14*^*C]2DG*: 2-deoxyglucose, and *AUC*: area under the curve.

Plasma insulin concentrations were measured by an Ultra-Sensitive Rat Insulin ELISA kit (#90060; Crystal Chem). Plasma non-esterified fatty acid (NEFA) levels were measured using a WAKO Diagnostics assay (#434-91795, #436-91995, and #4270-77000).

### 2.6 Immunoblotting

Mouse adipose tissue samples including epididymal, inguinal subcutaneous, and interscapular brown fat depots were homogenized in buffer containing 50 mM of Tris, 1 mM of EDTA, 1 mM of EGTA, 10% glycerol, 1% Triton x 100, 1 mM of DTT, 1 mM of PMSF, 1 × HALT protease inhibitor (#78430; Sigma), 50 mM of NaF, and 5 mM of NaPPi at a pH of 7.4 using 0.5 mm zirconium oxide beads (ZROB05; NextAdvance), centrifuged, and the supernatant was collected. The protein concentration was measured using a Pierce BCA Protein Assay kit (#23227; Thermo Fisher Scientific). Protein samples (20 μg) were run on a 10% SDS-page gel and transferred to nitrocellulose membranes (GE10600003; Sigma). The membranes were stained using Ponceau (P7170; Sigma) to normalize the samples. The following antibodies (Cell Signaling Technology) were used to detect proteins of interest: ILK (#3862), AKT (#9272), pAKT (#9271), SAPK/JNK (#9252), pSAPK/JNK (#9251), P38 (#9212), and pP38 (#9211) at a 1:1000 dilution.

### 2.7 Immunohistochemistry

Immunohistochemistry was performed on paraffin-embedded adipose tissue sections (5 μm) using the Dundee Tayside Biorepository Tissue Bank. The antibodies used were F4/80 (#MCA497R; AbD Serotec) and CD31 (#NBP1-49805; Novus Biologicals). Hematoxylin and eosin staining was used to quantify the cell size and number. Picro-Sirius Red staining was performed using Direct Red 80 (#365548; Sigma) and Picric acid (#P6744; Sigma). Images were captured using camera mounted on an AxioVision microscope (Zeiss Axioscope, Germany). The quantification was performed using Fiji and ImageJ. Cells were individually counted and measured for cell size and number. F4/80 and Sirius Red staining were quantified by the integrated intensity. CD31 was quantified by counting the positive structures.

### 2.8 Real-time PCR

RNA was extracted from the tissues using an RNeasy Mini kit (74104; Qiagen) and reversed transcribed into cDNA using Superscript II 10000U (18064014; Invitrogen). Quantitative real-time PCR was carried out using the following TaqMan probes from Applied Biosystems: IL-6 (Mm00446190_m1), TNF-α (Mm00443260_g1), IL-10 (Mm00439614_m1), IL-1b (Mm00434228_m1), PPARy (Mm00440904_m1), LPL (Mm00434764_m1), FASN (Mm00662319_m1), CD36 (Mm00432403_m1), UCP-1 (Mm01244861_m1), PGC-1α (Mm01208835_m1), CEBPα (Mm00514283_s1), HIF1α (Mm00468869_m1), GLUT4 (Mm01245502_m1), 18S (Hs99999901_s1), and β2M (Mm00437762_m1). All the data were normalized to the 18S or β2M gene expression and analyzed using the 2^-ΔΔCT^ method.

### 2.9 Liver triglycerides

Liver triglycerides were measured in ∼100 mg of tissue by colorimetric reactions using GPO (glycerol phosphate oxidase) triglyceride reagent (#T7532-500; Pointe Scientific).

### 2.10 Intraperitoneal glucose tolerance test (IPGTT)

The mice were weighed, individually housed, and fasted for 5 h prior to IPGTT. The baseline glucose level (t = 0) was taken for each mouse through tail vein sampling. The mice were injected intraperitoneally with 2 g/kg of 50% glucose and glucose levels measured over a 2-h period.

### 2.11 Flow cytometry

The epididymal fat and spleen were harvested from the mice and washed in cold sterile PBS to remove blood contamination. Cells were isolated as described by Orr et al., 2013 [23] and stained with APC α-mouse CD11b (#101211; BioLegend) and APC/Cy7 α-mouse F4/80 (#123117; BioLegend) for macrophages and PerCP/Cy5.5 α-mouse GR-1 (#108427; BioLegend) for neutrophils at a 1:200 dilution. DAPI staining (#D3571; Invitrogen) was used to quantify the live cell numbers.

### 2.12 Lipoprotein lipase activity

Lipoprotein lipase activity was determined by an Lipoprotein Lipase Activity Assay kit (Fluorometric) (ab204721; Abcam) in tissue lysates of the epididymal adipose tissue of the mice following the manufacturer's instructions.

### 2.13 Knockdown and knockout of ILK in 3T3-L1 cells

The 3T3-L1 cells were maintained in 10% FBS media (DMEM supplemented with 4.5 g/L of glucose, 1 mM of sodium pyruvate, 2 mM of glutamine, 20 mM of HEPES, 50 μg/ml of Pen/Strep, and 50 ml of FBS).

#### 2.13.1 sgRNA design and cloning

The BROAD Institute web portal was used to generate top sgRNA hits. The top five hits were used to create sgRNA oligos (forward and reverse). The oligos were duplexed and cloned into digested PX459 plasmid (1 ug of plasmid with 1 ul of the restriction enzyme *BbsI* (#R0539S; NEB)) by incubating the digested plasmid with the sgRNA and T4 ligase (#15224041; Thermo Fisher Scientific). After bacterial transformation, the positive clones were selected using AMP + plates. The positive colonies were cultured overnight at 37 °C and cloned PX459 plasmids were extracted using a QIAprep Spin Miniprep kit (#27104; Qiagen). The plasmids were sent for sequencing with LKO.1 forward primer (U6 promoter in the PX459 plasmid) to confirm the insertion of the sgRNA sequence.

#### 2.13.2 Cell transfection

Undifferentiated 3T3-L1 cells were cultured as previously described and transfected using Lipofectamine LTX with Plus Reagent (#A12621; Thermo Fisher Scientific). In a 12-well plate, 1 μg of cloned PX459 plasmids and 2 μl of Plus Reagent were added to 50 μl of Opti-MEM Reduced Serum Medium (#31985062; Invitrogen) in a fresh Eppendorf tube. In a separate tube, 3 μl of Lipofectamine LTX was added to 50 μl of Opti-MEM Reduced Serum Medium and both tubes were gently mixed and left to incubate at room temperature for 15 min. Then 50 μl of Lipofectamine LTX was gently added to the DNA/Plus Reagent complex and left to incubate for a further 30 min. Once the Lipofectamine LTX/DNA/Plus Reagent complex formed, 100 μl was added gently to the cells. Then, 400 μl of Opti-MEM was added to the cells and they were incubated for 6 h at 37 °C before adding 1 ml of 10% FBS media. After an overnight incubation, the media was replaced with 10% FBS media and the cells were left to recover for 24 h at 37 °C.

For cell selection, 2.5 ug/ml of puromycin was added to the cells for 48–72 h until all of the non-transfected control cells were dead. The media was then removed, the cells washed, and 20% FBS media (DMEM supplemented with 4.5 g/L of glucose, 1 mM of sodium pyruvate, 2 mM of glutamine, 20 mM of HEPES, 50 μg/ml of Pen/Strep, and 100 ml of FBS) added to allow the cells to recover and grow to confluency.

#### 2.13.3 Single-cell isolation and generation of stable cell lines

Once the cells reached confluency in a T150 flask, the cells were sent to be single-cell sorted into 96-well plates by FACS using the Core facility at the College of Life Sciences in Dundee. Once the cells reached confluency in the 96-well plates, they were grown up in T25 flasks, split, and analyzed by Western blotting for ILK protein expression. A portion of the cells was lysed in cell lysis buffer (25 mM of Tris–HCL, pH 7.4, 50 mM of NaF, 0.1 mM of NaCl, 1 mM of EDTA, 5 mM of EGTA, 1% Triton-X-100, 5 mM of NaPp, 92 mg/ml of sucrose, 0.1% mercaptoethanol, 1 mM of Na_3_VO_4_, 1 mM of benzamidine, and 0.1 M of PMSF) for genomic DNA extraction. The genomic DNA extraction was carried out following the protocol provided in a PureLink Genomic DNA Mini kit (#K182001; Thermo Fisher Scientific). Using primers to amplify the targeted area of the genome (5′ TCTCCGAGGTTCATTACC 3′ and 5′ GTTTGAAGTCAATACCGG 3’), the PCR products were cleaned using SureClean (#BIO-37047; Meridian Bioscience). Using a StrataClone PCR Cloning kit (#240205; Agilent), the PCR products were cloned into the pSC-A-amp/Kan vector to sequence individual alleles.

### 2.14 Oil Red O staining in the 3T3-L1 cells

A stock solution (3 mg/ml) of Oil Red O (#O0625; Sigma) was prepared in 100% isopropanol. The working solution was composed of a ratio of 3:2 of stock solution to dH_2_0. Once the cells fully differentiated, the media was removed, and the cells were washed with 500 μl of PBS and fixed in 400 μl of 4% PFA (#158127; Sigma) for 20 min at room temperature. The cells were washed with 1 × PBS twice followed by a wash with 60% isopropanol for 5 min and left to dry. Once the cells were completely dry, 400 μl of Oil Red O working solution was added to each well and incubated for 10 min. The Oil Red O was then removed, and the cells were washed four times with dH_2_0 to remove any excess stain. Once the water was removed and the cells were dry, the Oil Red O was eluted using 400 μl of 100% isopropanol and left for 1 h to ensure complete elution. The isopropanol was then collected for analysis by measuring the absorbance at 495 nm with an Envision 2104 Multilabel Plate Reader.

### 2.15 Statistical analysis

All the data were analyzed by unpaired Student's t-test or two-way ANOVA followed by Tukey's method for multiple comparisons where appropriate. The significance level was p < 0.05∗, p < 0.01∗∗, p < 0.005∗∗∗, and p < 0.001∗∗∗∗. All of the data are expressed as mean ± SEM.

## 3 Results

### 3.1 ILK protein expression increased in visceral adipose tissue of morbidly obese humans and diet-induced obese mice

**Figure 1:**
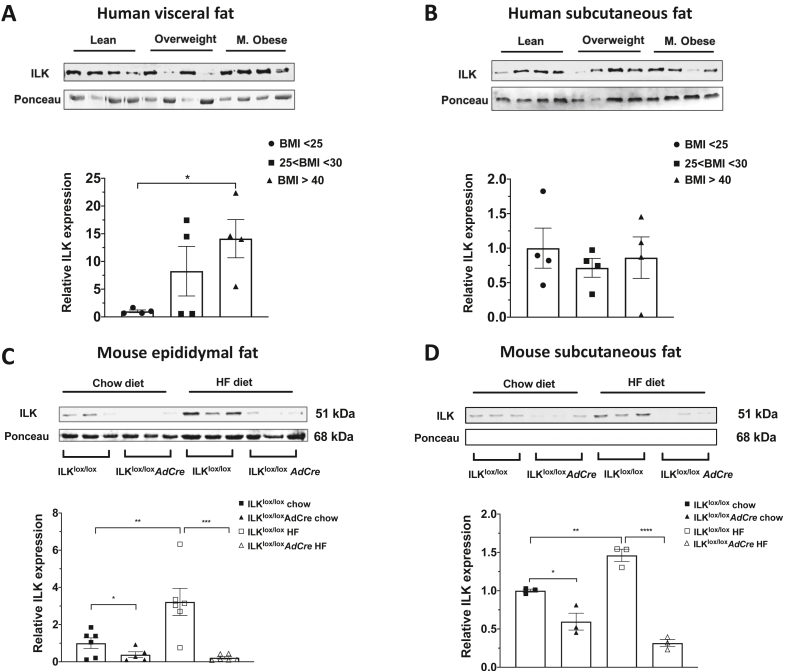
**ILK expression in human visceral and subcutaneous adipose tissue and murine epididymal and subcutaneous adipose tissue**. **(A and B)** ILK protein quantification by Western blotting in visceral and subcutaneous fat pads in humans normalized to lean individuals (n = 4). **(C and D)** Western blotting of epididymal and subcutaneous adipose lysates to quantify ILK expression in the chow and HF-fed ILK^lox/lox^ and ILK^lox/lox^*AdCre* mice and normalized to the ILK^lox/lox^ on the chow diet (n = 3–6). All of the data are represented by mean +/− SEM with significance: ∗p < 0.05, ∗∗p < 0.01, ∗∗∗p < 0.005, and ∗∗∗∗p < 0.001.

The protein expression of ILK was measured in visceral (vWAT) and subcutaneous (sWAT) adipose tissue in human subjects (anthropometric data of the human subjects are shown in Table S1). Although there was no change in the ILK expression in the sWAT of obese subjects, there was an increase in ILK expression in morbidly obese individuals compared to lean individuals in the vWAT (Figure 1A/B). Consistent with our findings in humans, ILK protein expression in epididymal fat (visceral fat equivalent in mice) also increased in the high-fat (HF) diet-fed obese wild-type mice (ILK^lox/lox^) compared with the lean ILK^lox/lox^ mice (Figure 1C). Interestingly, ILK protein expression was also found to be increased in the subcutaneous fat of the HF-fed ILK^lox/lox^ mice relative to lean ILK^lox/lox^ mice (Figure 1D).

### 3.2 Adipocyte-specific deletion of ILK decreased adiposity in obese mice without disrupting energy homeostasis

**Figure 2:**
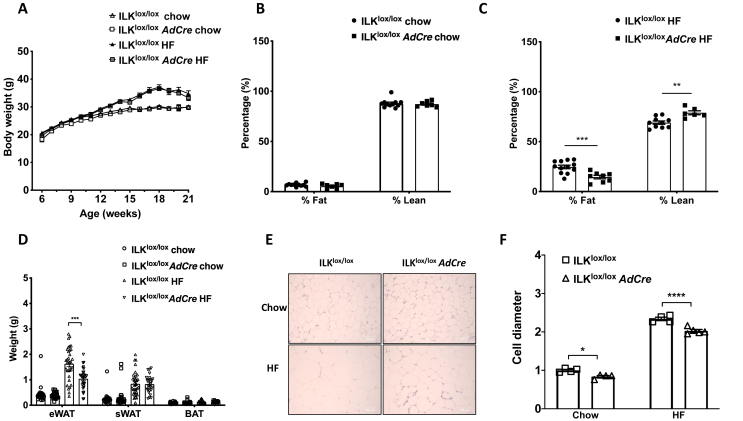
**Phenotypic differences between the ILK**^**lox/lox**^**and ILK**^**lox/lox**^***AdCre* mice**. (**A)** Body weights of the mice from ages 6 weeks–21 weeks on the chow and HF diets (n = 10–17). **(B and C)** Body composition of the chow and HF-fed mice expressed as the percentage of fat and lean mass at 21 weeks of age (n = 10–13). **(D)** Wet weights of fat depots from the chow and HF-fed mice (n = 16–30). **(E and F)** Hematoxylin and eosin staining in the eWAT to measure the cell diameter in the ILK^lox/lox^ and ILK^lox/lox^*AdCre* mice fed chow and HF diets normalized to the chow-fed ILK^lox/lox^ mice (n = 4–5). All of the data are represented by mean +/− SEM with significance: ∗p < 0.05, ∗∗p < 0.01, ∗∗∗p < 0.005, and ∗∗∗∗p < 0.001.

To investigate the pathophysiological role of ILK in obesity and insulin resistance, adipocyte-specific ILK knockout mice (ILK^lox/lox^*AdCre*) were studied. ILK protein expression in the whole adipose tissue lysates of the ILK^lox/lox^*AdCre* mice significantly decreased compared to the wild-type littermate ILK^lox/lox^ mice on both HF and chow diets regardless of fat depots (Figure 1C/D). Body weight, body composition, and wet weights of individual tissues were not different between the ILK^lox/lox^ and ILK^lox/lox^*AdCre* mice on the chow diet (Figure 2A/B and Fig. S1A). In contrast, on the HF diet, there was a decrease in the percent of fat mass and an increase in the percent of lean mass in the ILK^lox/lox^*AdCre* mice compared to the ILK^lox/lox^ mice despite no changes in total body weights (Figure 2A/C). Consistent with the reduction in the percent of fat mass, the epididymal fat (eWAT) tissue weight in the HF-fed ILK^lox/lox^*AdCre* mice was lower compared to the HF-fed ILK^lox/lox^ mice (Figure 2D), while tissue weights of sWAT, brown adipose tissue (BAT), and other non-fat tissues remained unchanged (Figure 2D and Figure S1B). A decreased percent of fat mass in the HF-fed ILK^lox/lox^*AdCre* mice was associated with a decrease in the adipocyte cell size in the eWAT of the ILK^lox/lox^*AdCre* mice compared to the ILK^lox/lox^ mice regardless of diet (Figure 2E/F).

Decreased adiposity is often associated with improved metabolic regulation [24], so we next assessed energy and glucose homeostasis in these mice. The RER, ambulatory activity, food intake, and energy expenditure were not significantly different between the two genotypes on either chow or HF diets (Figures S2A–H), although the mice on the HF diet showed a decrease in their overall RER with disruption to their circadian rhythm patterns as expected (Figure S2E).

### 3.3 Adipocyte-specific deletion of ILK improved glucose tolerance and the anti-lipolytic action of insulin on lipolysis, and increased insulin-stimulated glucose uptake in BAT of the obese mice

**Figure 3:**
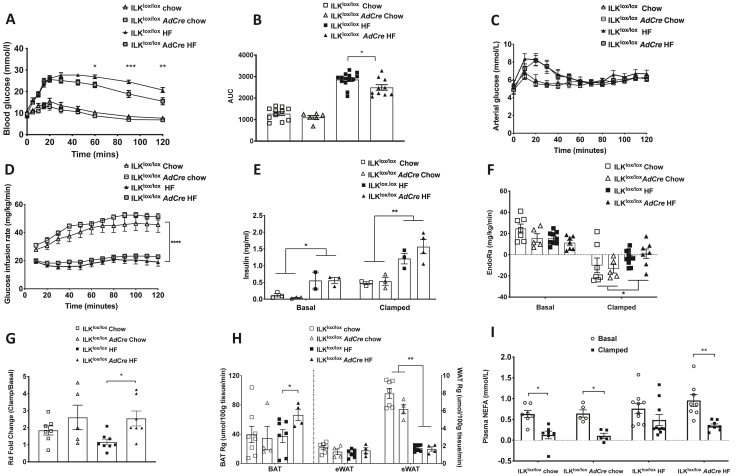
**Glucose homeostasis and insulin sensitivity in the ILK**^**lox/lox**^**and ILK**^**lox/lox**^***AdCre* mice**. **(A and B)** Venous glucose levels following intra-peritoneal glucose tolerance tests in the ILK^lox/lox^ and ILK^lox/lox^*AdCre* mice fed either chow or HF diets represented over time and by the area under the curve (AUC) (n = 6-13). **(C–I)** The hyperinsulinemic-euglycemic clamp in the ILK^lox/lox^ vs ILK^lox/lox^*AdCre* mice fed either chow or HF diets. **(C)** Arterial glucose over time in all four groups maintained at 6 mmoL/l over the course of the steady state of the hyperinsulinemic-euglycemic clamp (n = 5-9). **(D)** Variation in the glucose infusion rate to maintain the arterial glucose at a steady state in all four groups of mice (n = 5-9). **(E)** Insulin levels in basal and clamped plasma samples in all four groups of mice (n = 2-4). **(F)** EndoRa in basal and clamped plasma samples of the ILK^lox/lox^ vs ILK^lox/lox^*AdCre* mice on chow and HF diets (n = 5-9). **(G)** Rd in chow and HF diet-fed ILK^lox/lox^ vs ILK^lox/lox^*AdCre* mice (n = 5-8). **(H)** Rg metabolic index for tissue glucose uptake in eWAT, sWAT, and BAT (n = 4-8). **I:** Plasma non-esterified fatty acid (NEFA) levels at baseline (t = 0 min) and during the steady state of the clamp (80–120 min) (n = 5-10). All of the data are represented by mean +/− SEM with significance: ∗p < 0.05, ∗∗p < 0.01, ∗∗∗p < 0.005, and ∗∗∗∗p < 0.001.

Glucose tolerance was impaired in the HF-fed mice compared to the chow-fed mice, yet the HF-fed ILK^lox/lox^*AdCre* mice had improved glucose tolerance compared to the HF-fed ILK^lox/lox^ mice (Figure 3A/B). We next measured the insulin sensitivity using the hyperinsulinemic-euglycemic clamps (ICv). The arterial glucose levels were clamped at ∼6 mmol/L at the steady state of the ICv clamps (80–120 min) in all of the mice (Figure 3C). The glucose infusion rate of the chow-fed mice was significantly higher than the mice fed a HF diet, confirming the presence of whole-body insulin resistance (Figure 3D). There were no significant differences in glucose infusion rates between the genotypes regardless of diet. Plasma insulin concentrations were the same between the genotypes at the baseline and were equivalently increased during the ICv clamps (Figure 3E). HF diet feeding increased basal and clamp insulin levels in both genotypes. Basal EndoRa was not different between the genotypes or diets (Figure 3F). In the presence of insulin, EndoRa decreased in all of the groups of mice with greater reductions in the chow-fed mice compared to the HF-fed mice (Figure 3F). However, there were no differences in clamp EndoRa between the genotypes regardless of diet. The fold increase (clamp vs baseline) in Rd was the same between the chow-fed ILK^lox/lox^ and ILK^lox/lox^*AdCre* mice but increased in the HF-fed ILK^lox/lox^*AdCre* mice compared to the HF-fed ILK^lox/lox^ mice (Figure 3G). Consistent with the increased Rd in the HF-fed ILK^lox/lox^*AdCre* mice, the tissue-specific glucose uptake index (Rg) in the brown adipose tissue (BAT) was increased in the HF-fed ILK^lox/lox^*AdCre* mice compared with the HF diet-fed ILK^lox/lox^ mice (Figure 3H). Although the HF diet caused a reduction in the Rg in the eWAT and sWAT in the mice, no differences were seen in Rgs between the genotypes on either diet (Figure 3H). The basal levels of plasma NEFA were the same between the four groups of mice (Figure 3I). During the ICv clamps, the NEFA levels significantly decreased in the chow-fed mice in both genotypes. HF diet feeding in the ILK^lox/lox^ mice caused insulin resistance to suppress the arterial NEFA levels. However, this resistance was diminished in the HF-fed ILK^lox/lox^*AdCre* mice and therefore the ability of insulin to suppress the NEFA concentration improved. As NEFA is a surrogate marker of lipolysis, this finding suggested a greater suppression of adipocyte lipolysis in the HF-fed ILK^lox/lox^*AdCre* mice (Figure 3I). Collectively, these results suggested that under HF diet feeding, adipocyte-specific deletion of ILK improved insulin sensitivity by stimulating glucose utilization in BAT and inhibiting systemic lipolysis.

### 3.4 Improved insulin resistance in BAT of the HF-fed ILK^lox/lox^*AdCre* mice was associated with increased Akt phosphorylation and P38/JNK dephosphorylation

**Figure 4:**
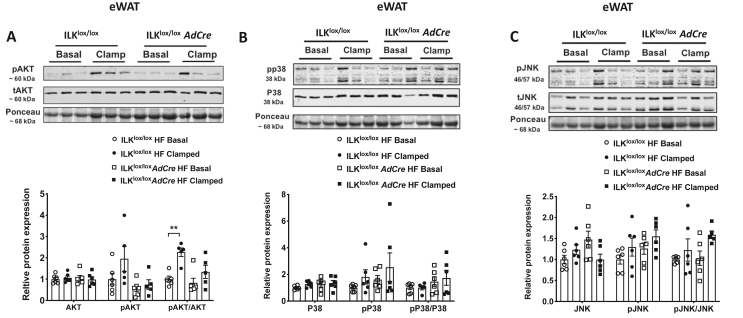
**Insulin and MAPK signaling in the eWAT of the basal and clamped HF diet-fed ILK**^**lox/lox**^**and ILK**^**lox/lox**^***AdCre* m**ice. Total protein and phosphorylated protein levels of AKT (A), p38 (B), and JNK (C) measured by Western blotting. The ratio of phosphorylated protein to total protein was normalized to ILK^lox/lox^ mice (n = 5-6). All of the data are represented by mean +/− SEM with significance: ∗∗p < 0.01.

**Figure 5:**
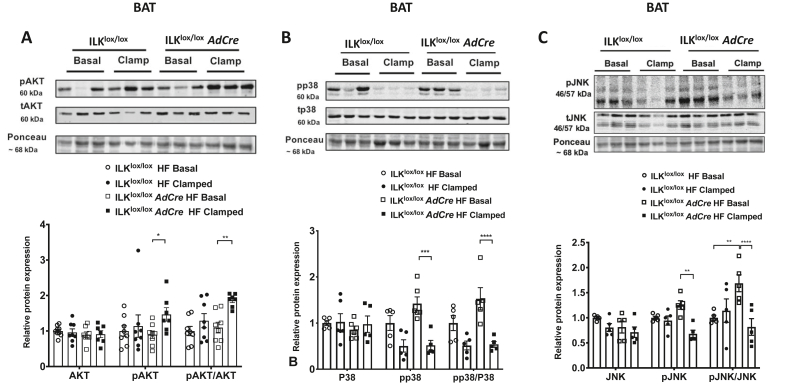
**Insulin and MAPK signaling in the BAT of the basal and clamped HF diet-fed ILK**^**lox/lox**^**and ILK**^**lox/lox**^***AdCre* mice**. Total protein and phosphorylated protein levels of AKT **(A)**, p38 **(B)**, and JNK **(C)** were measured by Western blotting. The ratio of phosphorylated protein to total protein was normalized to the ILK^lox/lox^ mice (n = 5-8). All of the data are represented by mean +/− SEM with significance: ∗p < 0.05, ∗∗p < 0.01, ∗∗∗p < 0.005, and ∗∗∗∗p < 0.001.

To further understand the underlying mechanisms for altered insulin action, insulin signaling was studied in eWAT and BAT. In eWAT, the pAKT/AKT ratio increased in the clamped tissues of the HF diet-fed ILK^lox/lox^ mice compared with those in basal 5-h-fasted basal tissues. This increase was absent in the HF-fed ILK^lox/lox^*AdCre* mice (Figure 4A). The mitogen-activated protein kinase (MAPK) pathway including p38 and JNK showed no response to insulin in either genotype in eWAT (Figure 4B/C). In contrast to eWAT, both pAKT and pAKT/AKT ratios were increased in BAT of the HF-fed ILK^lox/lox^*AdCre* mice in response to insulin stimulation. No response of pAKT and pAKT/AKT ratios to insulin was observed in the HF-fed ILK^lox/lox^ mice (Figure 5A). Interestingly, unlike in eWAT, both p38 and JNK showed a decrease in phosphorylation in clamped BAT compared to basal BAT in the HF-fed ILK^lox/lox^*AdCre* mice. No such effect was seen in the HF-fed ILK^lox/lox^ mice (Figure 5B/C). The pp38/p38 and pJNK/JNK ratios also significantly decreased in insulin-clamped tissues of the HF-fed ILK^lox/lox^*AdCre* mice relative to basal tissues, with no such differences in the HF-fed ILK^lox/lox^ mice (Figure 5B/C). Furthermore, the increase in insulin-stimulated glucose uptake in BAT of the HF-fed ILK^lox/lox^*AdCre* mice was not associated with changes in gene expression of UCP-1 or GLUT4 (Figure S3).

### 3.5 Improved anti-lipolytic response of insulin in the HF-fed ILK^lox/lox^*AdCre* mice was associated with increased CD31 expression without changes in fibrosis or inflammation in eWAT

**Figure 6:**
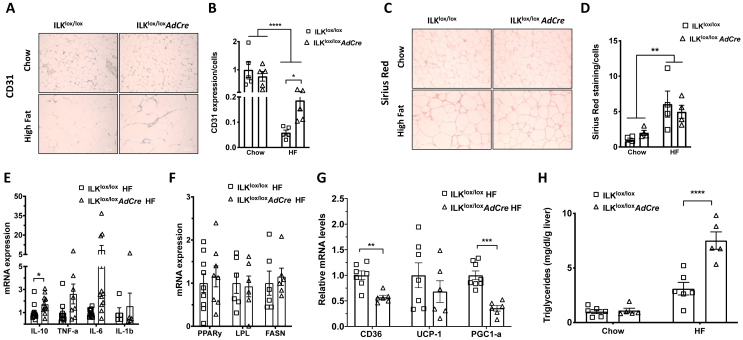
**Vascularization, fibrosis, and mRNA expression in the epididymal fat of the ILK**^**lox/lox**^**and ILK**^**lox/lox**^***AdCre* mice**. **(A and B)** Immunohistochemical analysis of the vascularization in eWAT using CD31 antibody in the ILK^lox/lox^ and ILK^lox/lox^*AdCre* mice fed chow and HF diets normalized to the chow-fed ILK^lox/lox^ mice (n = 5). **(C and D)** Picro-Sirius Red staining of eWAT in the ILK^lox/lox^ and ILK^lox/lox^*AdCre* mice fed chow and HF diets normalized to the chow-fed ILK^lox/lox^ mice (n = 4). **(E**–**G)** q-PCR analysis of gene expression in eWAT of the HF diet-fed ILK^lox/lox^ and ILK^lox/lox^*AdCre* mice normalized to the HF-fed ILK^lox/lox^ (n = 6-9). **H**: Hepatic triglyceride levels in the chow- and HF-fed ILK^lox/lox^ and ILK^lox/lox^*AdCre* mice normalized to the chow-fed ILK^lox/lox^ (n = 5-6). All of the data are represented by mean +/− SEM with significance: ∗p < 0.05, ∗∗p < 0.01, ∗∗∗p < 0.005, and ∗∗∗∗p < 0.001.

To further investigate how deletion of ILK decreased fat mass in eWAT of the HF-fed mice, we examined the cell morphology and endocrine function of eWAT. The CD31 expression decreased in the HF-fed mice compared to the chow-fed mice in both genotypes (Figure 6A/B). The ILK^lox/lox^*AdCre* mice challenged on the HF diet showed an increase in CD31 expression compared to the HF-fed ILK^lox/lox^ mice, suggesting improved vascularization (Figure 6A/B). Fibrosis of the eWAT as detected by Picro-Sirius Red staining was also increased in the HF-fed mice compared to the chow-fed mice (Figure 6C/D). However, there were no differences between genotypes regardless of diet (Figure 6C/D). Similarly, HF diet feeding increased eWAT hypoxia as shown by the increased gene expression of HIF1α in both genotypes, but no differences were observed between genotypes regardless of diet (Figure S4).

We next assessed the inflammatory status of the HF-fed ILK-deficient mice. Although the gene expression of IL-10 significantly increased in the HF-fed ILK^lox/lox^*AdCre* mice compared with the HF-fed ILK^lox/lox^ mice, genetic deletion of ILK in adipocytes did not affect the gene expression of the inflammatory markers including TNF-α, IL-6, or IL-1b (Figure 6E). Furthermore, macrophage and neutrophil infiltration in the eWAT and spleen was measured by flow cytometry and there were no differences observed between the genotypes (Figure S5A/B). This was supported by the demonstration that the F4/80 expression in eWAT of the HF-fed mice increased relative to the chow-fed mice, but there was no difference in macrophage infiltration between the ILK^lox/lox^ and ILK^lox/lox^*AdCre* mice on either diet (Figure S5C/D). Taken together, these data suggested that adipocyte-specific deletion of ILK did not significantly change the inflammatory status of the mice after HF diet feeding, with similar levels of immune cell infiltration, but was characterized by an increased expression of IL-10 that could lead to an increase in its secretion.

### 3.6 Decreased fat mass in the HF-fed ILK^lox/lox^*AdCre* mice may have been attributed to decreased fatty acid uptake to adipocytes with fat redistributed to the liver

Decreased fat mass in the HF-fed ILK^lox/lox^*AdCre* mice did not seem to be attributed to changes in gene expression of PPARγ, LPL, or FASN in eWAT (Figure 6F). Likewise, LPL activity in eWAT was also the same between the HF-fed ILK^lox/lox^*AdCre* and ILK^lox/lox^ mice (Figure S6). In contrast, the gene expression of CD36, fatty acid translocase, and PGC-1α, a mitochondrial biogenesis marker, significantly decreased in the HF-fed ILK^lox/lox^*AdCre* mice compared with the HF-fed ILK^lox/lox^ mice, while the UCP-1 expression was not altered (Figure 6G). These results suggested that decreased fat mass in the HF-fed ILK-deficient mice may have been attributed to decreased fatty acid uptake into adipocytes. To further examine where the fat went, we assessed the liver triglyceride content and found that liver triglyceride significantly increased in the HF-fed ILK^lox/lox^*AdCre* mice compared with the HF-fed ILK^lox/lox^ mice despite no differences between the genotypes in the chow-fed lean mice (Figure 6H).

### 3.7 Reduction/deletion of ILK impaired adipogenesis in 3T3-L1 cells

**Figure 7:**
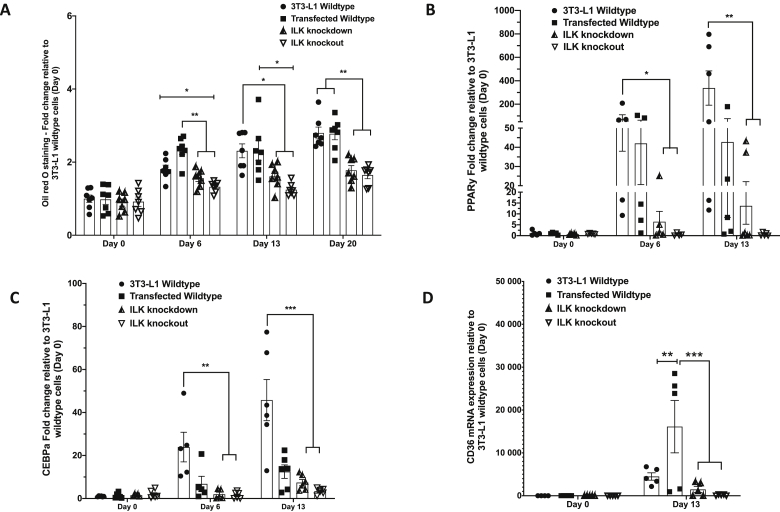
**Lipid accumulation and PPARγ, CEBPα, and CD36 mRNA expression in stable ILK 3T3-L1 cells.** The stable ILK 3T3-L1 knockdown and knockout cell lines were generated using the CRISPR-Cas9 system. **(A)** Oil Red O staining of naïve 3T3-L1 wild-type cells, transfected wild-type cells, ILK knockdown cells, and ILK knockout cells (n = 7). Data were normalized to the naïve 3T3-L1 wild-type cells on day 0 (prior to undifferentiation). **(B**–**D)** mRNA expression of PPARγ, CEBPα, and CD36 respectively in naïve 3T3-L1 wild-type cells, transfected wild-type cells, ILK knockdown cells, and ILK knockout cells (n = 5–6). Data were normalized to the naïve wild-type cells on day 0 (prior to undifferentiation). All of the data are represented by mean +/− SEM with significance: ∗p < 0.05, ∗∗p < 0.01, ∗∗∗p < 0.005, and ∗∗∗∗p < 0.001.

To further understand the role of ILK in adipogenesis and lipid metabolism in adipocytes, we generated ILK knockdown (KD) and knockout (KO) 3T3-L1 cells using the CRISPR-Cas9 technique (Figure S7). Biallelic sequencing data confirmed that the transfected wild-type cells (tWT) had identical sequences with the naïve 3T3-L1 cells, the KD cells had mutations in one allele with a 4bp deletion, and the KO cells had mutations on both alleles with a 5bp and 1bp deletion, respectively (Figure S8). Upon stimulation for differentiation, lipid accumulation measured by Oil Red O staining decreased by both KD and KO of ILK (Figure 7A). Gene expression of adipogenic markers PPARɣ and C/EBPα decreased in ILK KD and KO cells compared with 3T3-L1-naïve cells (Figure 7B/C). Moreover, gene expression of CD36 also significantly decreased in the ILK KD and KO cells compared with the tWT controls (Figure 7D). These results suggested that ILK reduction impaired adipogenesis in the 3T3-L1 cells.

## 4 Discussion

**Figure 8:**
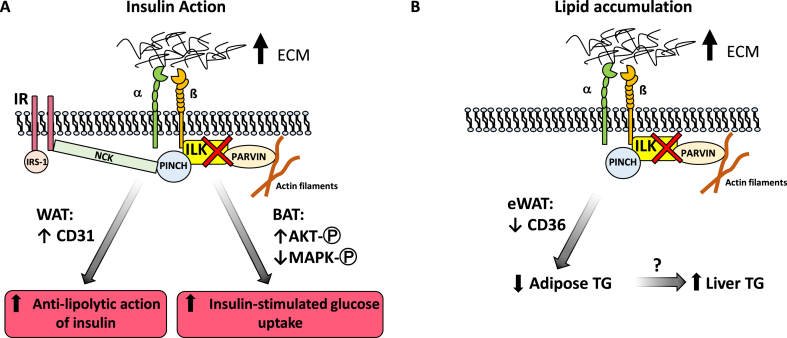
**Proposed working model of how deletion of ILK in adipocytes affected insulin sensitivity and lipid accumulation**. (**A)** The deletion of ILK in adipocytes of WAT resulted in improved vascularization, which may contribute to improving the anti-lipolytic activity of insulin. In contrast, deletion of ILK in adipocytes of BAT increased insulin-stimulated AKT phosphorylation and decreased p38 and JNK phosphorylation resulting in improved glucose uptake in response to insulin. (**B)** It is proposed that deletion of ILK in adipocytes of WAT resulted in a decrease in CD36 gene expression and subsequent decrease in NEFA uptake into adipocytes and decreased adipose triglyceride (TG) levels. This decrease in NEFA uptake resulted in an increase in hepatic lipid accumulation.

ECM remodeling in adipose tissue is closely associated with developing insulin resistance in obesity [3]. Many previous studies from our group have proven a key role of the ECM-integrin-ILK pathway in the pathogenesis of diet-induced insulin resistance in skeletal muscle and the liver [14,15,25,26]. In this study, we for the first time demonstrated that ILK in adipocytes is pivotal for regulating obesity-associated adipocyte hypertrophy and insulin resistance, but not inflammation. In the HF diet-induced obese mice, we showed that adipocyte-specific deletion of ILK caused smaller epididymal fat pads, decreased percent fat mass, and partially ameliorated insulin resistance in adipose tissue by increasing glucose uptake in BAT and enhancing the inhibitory action of insulin on lipolysis as measured by plasma NEFA levels as a surrogate marker. Our studies also provide mechanistic insight in that increased vascularization in eWAT may contribute to increased insulin sensitivity for inhibiting lipolysis. Moreover, increased insulin-stimulated AKT phosphorylation and P38 and JNK dephosphorylation may contribute to increased insulin sensitivity for stimulating glucose uptake in BAT (Figure 8A). Furthermore, it is hypothesized that decreased fat storage in epididymal adipose tissue was due to decreased fatty acid uptake into adipocytes through CD36 at the expense of fatty liver (Figure 8B). While our current study demonstrated a novel role of adipocyte ILK in adipose function and insulin resistance, it is important to highlight the study's limitations. Only male mice were used in this study, which caused sex bias and constrained the generalizability of the data in females. Despite an initial power calculation for the sample size of the animals (n = 8 with α = 0.05 and power = 0.8), the n number of some of the analyses (glucose uptake) was low due to technical difficulties and therefore presents the possibility of underpower.

Our results from adipocyte-specific deletion of ILK were consistent with previous studies in muscle and liver, suggesting an important role of the ECM-integrin-ILK pathway in promoting insulin resistance in insulin-sensitive tissues of mice on a HF diet [14,15]. Likewise, upon insulin stimulation, the presence of ILK restrained insulin signaling among various tissues including skeletal muscle [14,15] and BAT in the present study. ILK had no catalytic activity. Therefore, it is proposed that the effects of ILK deletion may have been through the loss of the ILK-PINCH-Parvin (IPP) complex. Specifically, PINCH is associated with the insulin receptor signaling pathway by binding to Nck2, which in turn interacts with insulin receptor substrate-1 (IRS-1) [27]. Graae et al. recently demonstrated that overexpression of ADAMTS9, a secreted metalloproteinase, decreases insulin signaling and action at least partially through altering integrin signaling with increased ILK and PINCH expression [28]. These results suggest that ADAMTS9 may also be a potential link between insulin signaling and the IPP complex. Parvin on the other hand mediates cytoskeletal dynamics by binding to F-actin and is proposed to regulate mitochondrial energetics and subsequent glucose metabolism through Rac1 and cofilin [29]. A potential role of Parvin in the context of insulin action has not been elucidated. It was also intriguing to see that deletion of ILK specifically in the skeletal muscle, liver, or adipose tissue had beneficial effects on systemic insulin resistance or glucose intolerance. While the skeletal muscle, liver, and adipose tissue are major organs contributing to whole-body glucose metabolism, they also secret circulating factors to execute paracrine function and regulate insulin response in other tissues. Indeed, deletion of ILK in skeletal muscle increases IL-6 gene expression in the muscle [14] and deletion of ILK in adipocytes increases IL-10 gene expression in adipose tissue. It would be interesting to see whether circulating levels of these cytokines are affected and their potential impact on insulin response.

In contrast to the emerging role of ILK in promoting obesity-associated insulin resistance, a recent study by Hatem-Vaquero et al. showed that global deletion of ILK in adulthood in mice caused impaired glucose and insulin tolerance on a chow diet, with a reduction in the expression and membrane presence of GLUT4 in the insulin-sensitive peripheral tissues [30]. Their study suggested a critical role of ILK in maintaining glucose homeostasis under normal physiological conditions. Our current study, however, suggested that ILK expression specifically in adipocytes was not required for the maintenance of glucose homeostasis in the chow-fed mice, with unchanged glucose tolerance and insulin sensitivity assessed by the insulin clamps. It was likely that a ubiquitous deletion of ILK in the study by Hatem-Vaquero et al. explained the discrepancies in the metabolic phenotype from our tissue-specific ILK-deficient mice [30].

It was significant to observe the remarkable effect of ILK deletion in reducing adipocyte size and diet-induced adiposity in the mice. Although this did not seem to be associated with changes in the gene expression of PPARγ, FASN, or LPL, it is possible that the difference in lipid accumulation in adipocytes was due to decreased uptake of exogenous lipids. In adipocytes, CD36, FABPpm, Caveolin 1, and FATP1 are known to be involved in the uptake of free fatty acids into cells [31]. Consistent with this concept, we observed that CD36 gene expression decreased in the HF-fed ILK-deficient mice. Few current reports support the role of ILK in fatty acid uptake. The IPP complex is critical to cytoskeletal dynamics that are necessary for protein translocation. It is possible that deletion of ILK would impair translocation of CD36 to the plasma membrane and subsequently decrease lipid uptake in adipocytes. Furthermore, our data in the 3T3-L1 cells showed evidence that ILK is essential for adipogenesis and CD36 gene expression in adipocytes. Nevertheless, decreased adipocyte hypertrophy in the adipocyte-specific ILK-deficient mice was consistent with decreased hepatic lipid accumulation in mice lacking ILK specific in hepatocytes [15], with the most prominent effect in the presence of dietary lipid excess. Decreased PGC-1α expression highlighted a potential role of ILK in mitochondrial biogenesis and fatty acid oxidation, which could also contribute to lipid metabolism in adipocytes. However, controversy persists over the effects of PGC-1α on the OXPHOS system, fatty acid transport, and fatty acid oxidation in adipocytes [[32], [33], [34]].

One of the most interesting findings was that increased ILK protein expression observed in eWAT of the HF diet-fed mice mirrored the increase in the visceral adipose tissue of morbidly obese humans. It is important to highlight that this diet-induced increase in ILK protein was absent in the adipose tissue of the adipocyte-specific ILK knockout mice, indicating that this increase was likely driven by the adipocytes themselves. The increase in ILK protein was maintained in the sWAT of the HF-fed mice, but absent in the human sWAT. Despite many similarities between white adipose depots from rodents and humans, notable differences emerge related to fat deposition and function [35]. In humans, increased visceral adipose tissue is associated with insulin resistance, dyslipidemia, and type 2 diabetes, while subcutaneous adipose tissue is associated with preserved insulin sensitivity and mitigates risk of metabolic disorders [[36], [37], [38], [39]]. Moreover, sWAT in humans is heterogeneous, having different physiological effects depending on the depot location, while rodent fat depots do not harbor the complexity of human sWAT [35]. Our results that ILK increased in vWAT but not sWAT of morbidly obese humans support the hypothesis that ILK in visceral fat plays a pathological role in obesity-related insulin resistance. The physiological and functional differences between sWAT from humans and mice may explain the differential regulation of ILK expression in sWAT between obese humans and mice. Moreover, our results suggest that the ECM-integrin-ILK pathway contributes to insulin resistance in mice, which may also apply to adipose tissue in morbidly obese humans.

It is interesting that p38 and JNK were dephosphorylated during insulin stimulation in BAT, but not in eWAT of the HF-fed mice. Although our results in eWAT were consistent with previous studies showing that insulin failed to induce any detectable activation of p38 MAPK in mouse primary adipocytes [40], dephosphorylation of p38 by insulin in BAT has not been shown. In 3T3-L1 adipocytes, however, activation of MAPKs including ERK, JNK, and P38 has been shown to reduce tyrosine phosphorylation of insulin receptor and IRS, decrease glucose transporter GLUT4, and induce insulin resistance [41]. Moreover, pharmacological inhibition of p38 MAPK improves insulin-stimulated uptake of glucose and increases protein levels of GLUT4 in 3T3-L1 adipocytes [42]. These studies were consistent with our results that increased p38/JNK dephosphorylation by insulin in the BAT of the ILK-deficient mice was accompanied by an increase in insulin-stimulated glucose uptake. These results were also consistent with our previous studies in muscle-ILK-deficient mice where improved insulin-stimulated muscle glucose uptake was accompanied by decreased p38 activation [14]. Our results showed that the gene expression of GLUT4 in BAT of the ILK-deficient mice was not altered. Yet it cannot rule out that protein expression of GLUT4 and/or membrane translocation of GLUT4 may have been affected by ILK deletion in BAT. Therefore, the hypothesis that in BAT, deletion of ILK decreases phosphorylation of p38 and JNK resulting in improved insulin-stimulated glucose uptake due to an increase in GLUT4 expression and translocation needs further investigation.

Neural innervation of adipose tissue is critical for metabolic processes including adipogenesis, lipolysis, and thermogenesis [43]. Vascularization and innervation are often linked and promote each other. Indeed, CD31 expression increased in the eWAT of the HF-fed ILK-deficient mice. It is therefore postulated that the effects of adipocyte ILK deletion on adipose function and insulin response may be centrally mediated through brain-adipose communication. The brain-adipose axis can also apply to the BAT where the sympathetic drive plays a key role in thermogenic activity. The effects of adipocyte ILK on vascularization and innervation of both eWAT and BAT are potentially important and remain to be studied.

## 5 Conclusion

In conclusion, our data showed that deletion of ILK in adipocytes had beneficial effects on metabolic regulation including decreasing adiposity and improving the anti-lipolytic action of insulin and insulin-stimulated glucose uptake in brown adipose tissue of obese insulin-resistant mice. In addition, our data provide mechanistic insight for underlying mechanisms showing the role of ILK in adipocytes in regulating the insulin and MAPK signaling pathways. Most importantly, by showing a similar increase in ILK expression in visceral adipose tissue of obese humans, our research is translational and may be relevant in a clinical setting. These data together with earlier studies in skeletal muscle and liver highly suggest that pharmacological disruption of ILK interactions would have metabolic beneficial effects on improving insulin sensitivity and provide promising therapeutic potential for diabetes and metabolic disorders. However, the potential role of ILK in decreasing fatty acid uptake through CD36 needs to be further investigated before its therapeutic consideration as it caused fatty liver in the current study.

## Funding

This work was supported by Diabetes UK 15/0005256 (LK, RJM and MLJA), European Commission Marie Curie International Incoming Fellowship 625119 (LK), and funding from the Diabetes Research and Wellness Foundation (LK) and TENOVUS Scotland (LK). This study was also supported by the Vanderbilt Mouse Metabolic Phenotyping Center (DK059637; DW). ABL is supported by a PhD scholarship from the Carnegie Trust. Human adipose tissue collections were supported by grants from the Medical Research Council MR/K010271/1 (RHS) and Chief Scientist Office SCAF/17/02 (RHS). The authors acknowledge the financial support of NHS Research Scotland (NRS) through the Edinburgh Clinical Research Facility.

## Author contributions

ABL and LK contributed to the experimental design, researched the data, contributed to the discussion and data interpretation, and wrote the manuscript. AH, XW, CL, and CKH researched the data and reviewed and edited the manuscript. RJM, RS, MLJA, and DHW contributed to the discussion and data interpretation and reviewed and edited the manuscript. All of the authors approved the manuscript's final version. The authors received no editorial assistance. LK is the guarantor of this work and, as such, had full access to all of the data and takes responsibility for the data integrity and accuracy of the data analysis.
