# Supplementary material for: Adipocyte integrin-linked kinase plays a key role in the development of diet-induced adipose insulin resistance in male mice
Source: Mol Metab. 2021 Feb 26;49:101197. doi: 10.1016/j.molmet.2021.101197 (PMC8027775; doi:10.1016/j.molmet.2021.101197)
Supplement: Multimedia component 1 [file mmc1.pptx]

## Slide 1
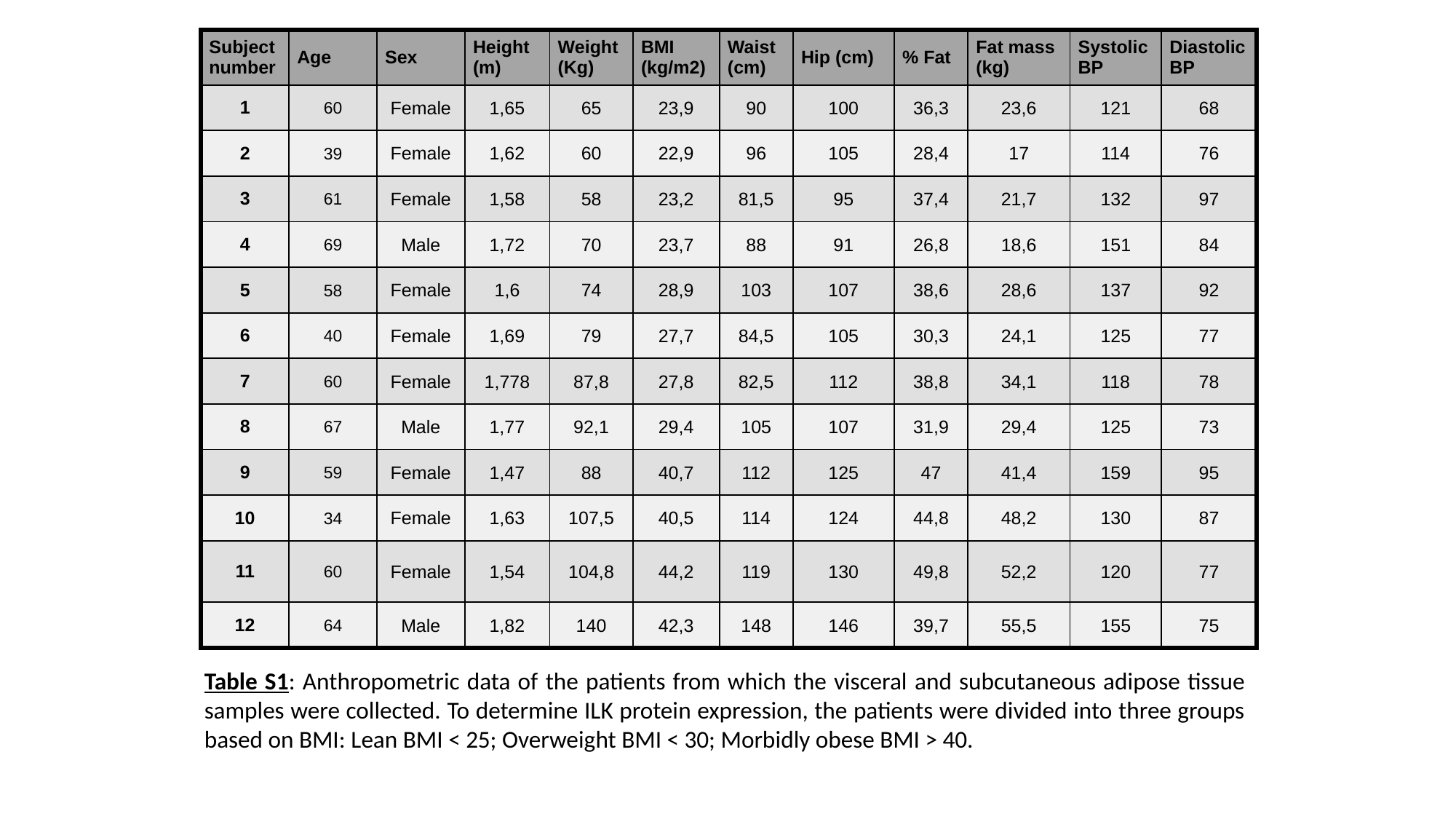

| Subject number | Age | Sex | Height (m) | Weight (Kg) | BMI (kg/m2) | Waist (cm) | Hip (cm) | % Fat | Fat mass (kg) | Systolic BP | Diastolic BP |
| --- | --- | --- | --- | --- | --- | --- | --- | --- | --- | --- | --- |
| 1 | 60 | Female | 1,65 | 65 | 23,9 | 90 | 100 | 36,3 | 23,6 | 121 | 68 |
| 2 | 39 | Female | 1,62 | 60 | 22,9 | 96 | 105 | 28,4 | 17 | 114 | 76 |
| 3 | 61 | Female | 1,58 | 58 | 23,2 | 81,5 | 95 | 37,4 | 21,7 | 132 | 97 |
| 4 | 69 | Male | 1,72 | 70 | 23,7 | 88 | 91 | 26,8 | 18,6 | 151 | 84 |
| 5 | 58 | Female | 1,6 | 74 | 28,9 | 103 | 107 | 38,6 | 28,6 | 137 | 92 |
| 6 | 40 | Female | 1,69 | 79 | 27,7 | 84,5 | 105 | 30,3 | 24,1 | 125 | 77 |
| 7 | 60 | Female | 1,778 | 87,8 | 27,8 | 82,5 | 112 | 38,8 | 34,1 | 118 | 78 |
| 8 | 67 | Male | 1,77 | 92,1 | 29,4 | 105 | 107 | 31,9 | 29,4 | 125 | 73 |
| 9 | 59 | Female | 1,47 | 88 | 40,7 | 112 | 125 | 47 | 41,4 | 159 | 95 |
| 10 | 34 | Female | 1,63 | 107,5 | 40,5 | 114 | 124 | 44,8 | 48,2 | 130 | 87 |
| 11 | 60 | Female | 1,54 | 104,8 | 44,2 | 119 | 130 | 49,8 | 52,2 | 120 | 77 |
| 12 | 64 | Male | 1,82 | 140 | 42,3 | 148 | 146 | 39,7 | 55,5 | 155 | 75 |
Table S1: Anthropometric data of the patients from which the visceral and subcutaneous adipose tissue samples were collected. To determine ILK protein expression, the patients were divided into three groups based on BMI: Lean BMI < 25; Overweight BMI < 30; Morbidly obese BMI > 40.

## Slide 2
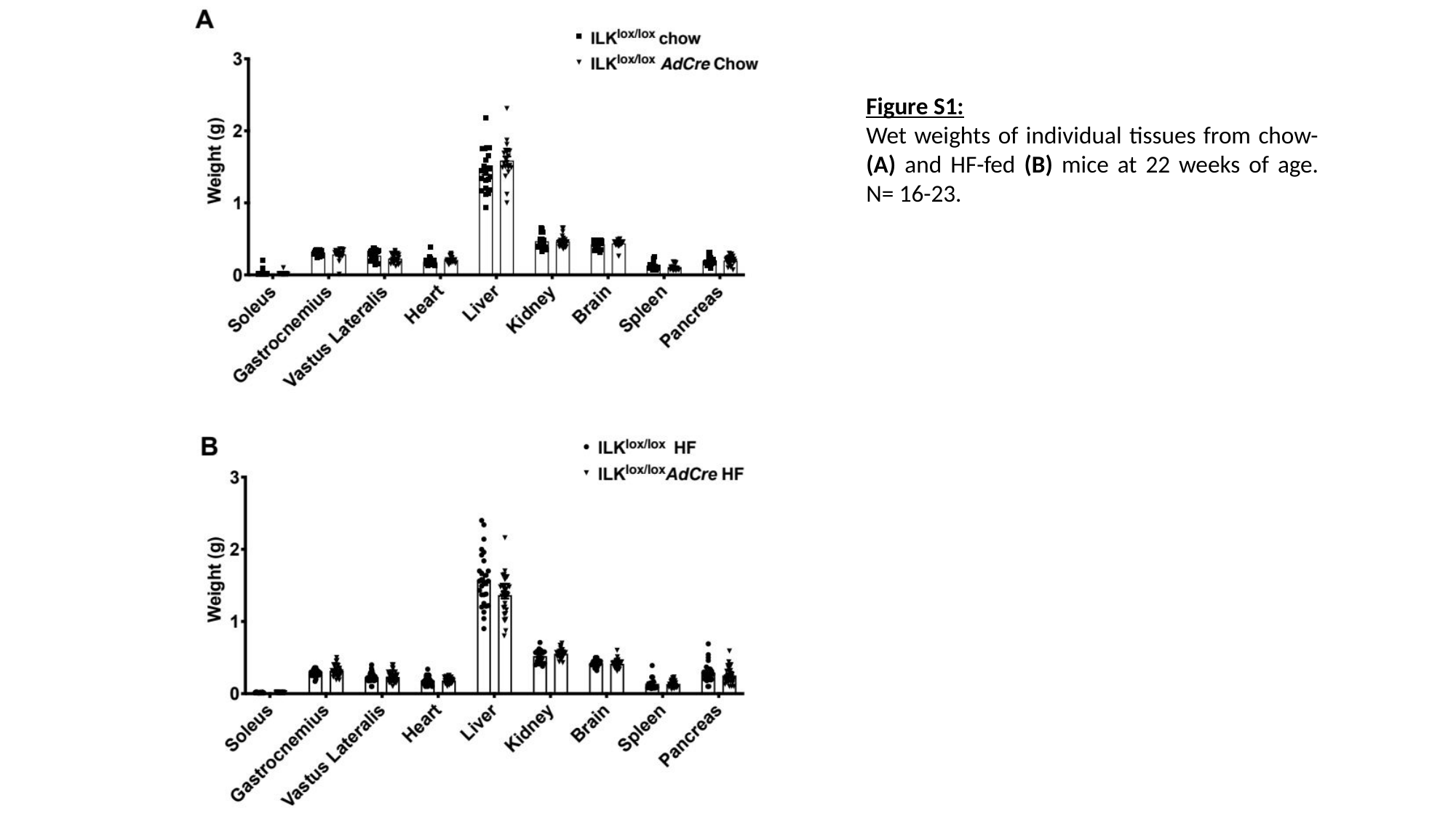

Figure S1:
Wet weights of individual tissues from chow- (A) and HF-fed (B) mice at 22 weeks of age. N= 16-23.

## Slide 3
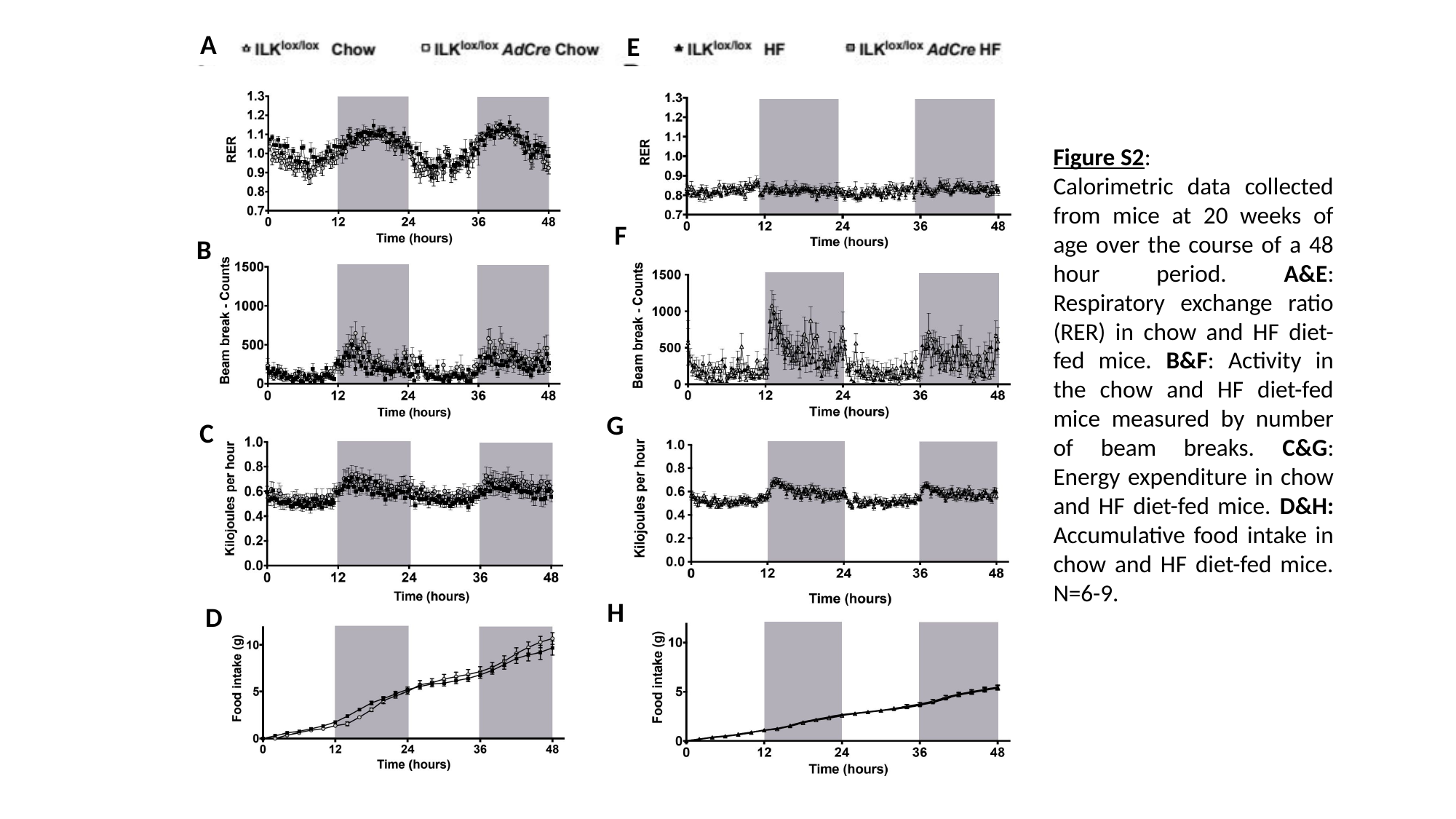

A
E
F
B
G
C
H
D
Figure S2:
Calorimetric data collected from mice at 20 weeks of age over the course of a 48 hour period. A&E: Respiratory exchange ratio (RER) in chow and HF diet-fed mice. B&F: Activity in the chow and HF diet-fed mice measured by number of beam breaks. C&G: Energy expenditure in chow and HF diet-fed mice. D&H: Accumulative food intake in chow and HF diet-fed mice. N=6-9.

## Slide 4
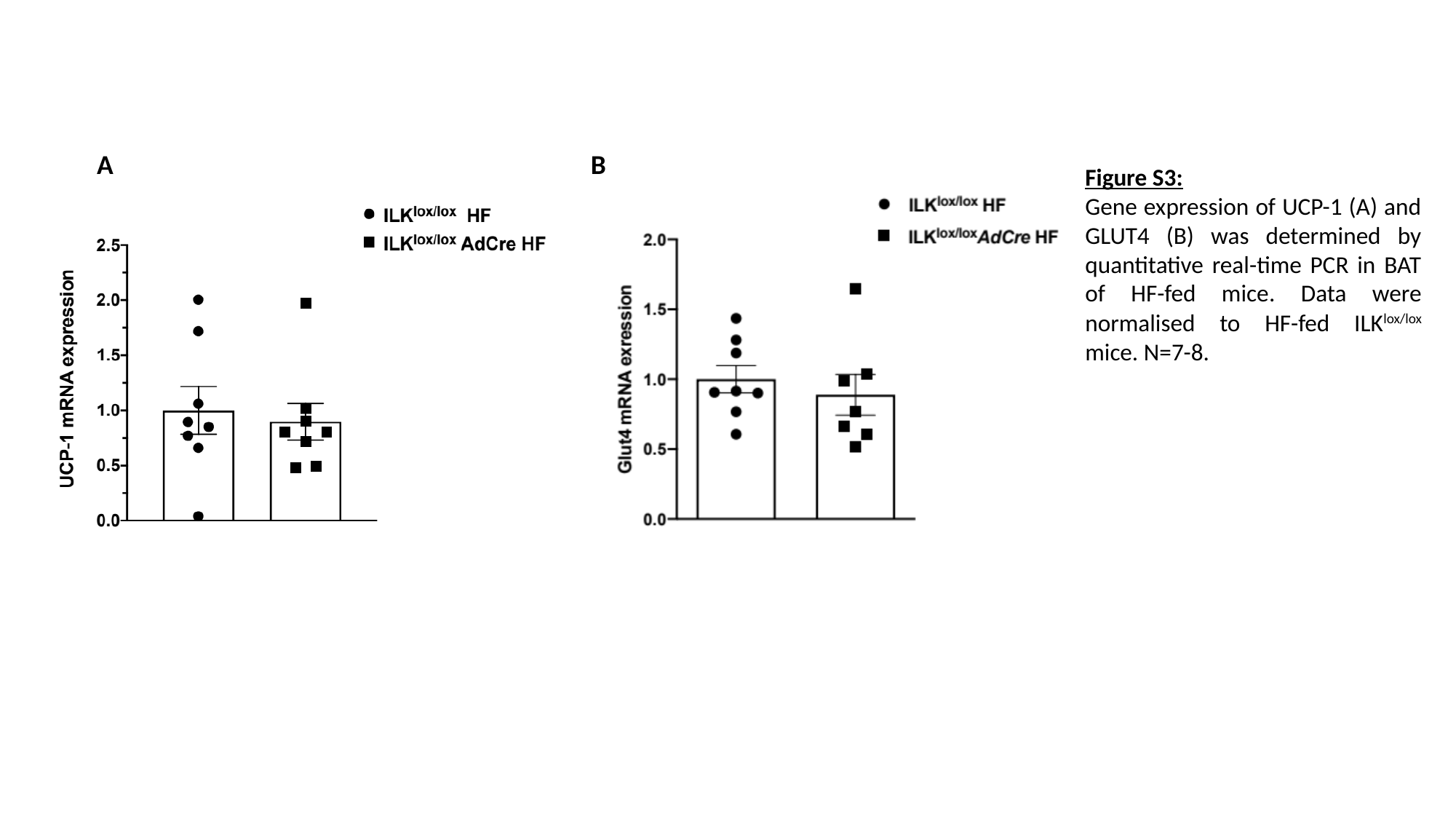

A
B
Figure S3:
Gene expression of UCP-1 (A) and GLUT4 (B) was determined by quantitative real-time PCR in BAT of HF-fed mice. Data were normalised to HF-fed ILKlox/lox mice. N=7-8.

## Slide 5
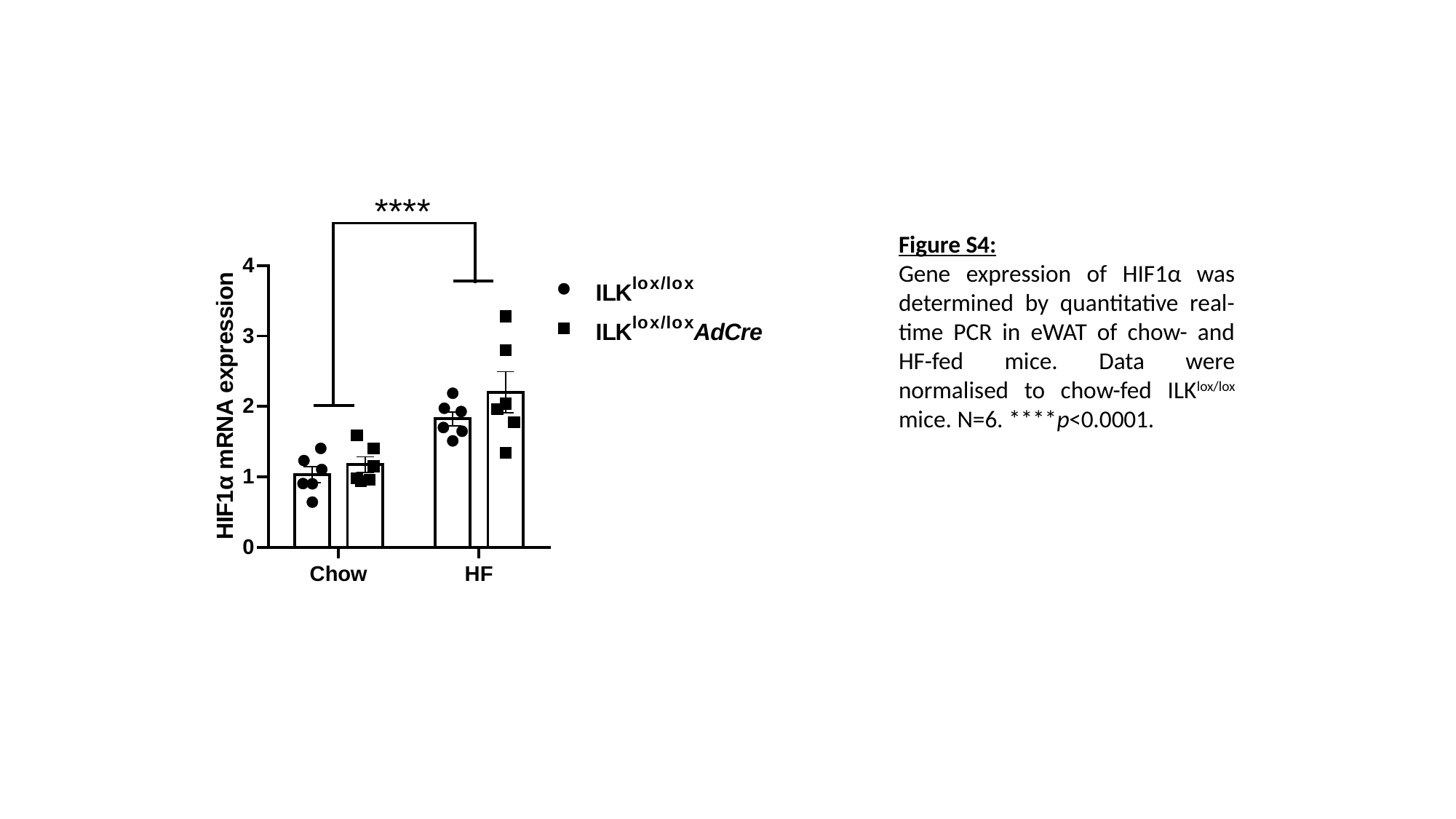

Figure S4:
Gene expression of HIF1α was determined by quantitative real-time PCR in eWAT of chow- and HF-fed mice. Data were normalised to chow-fed ILKlox/lox mice. N=6. ****p<0.0001.

## Slide 6
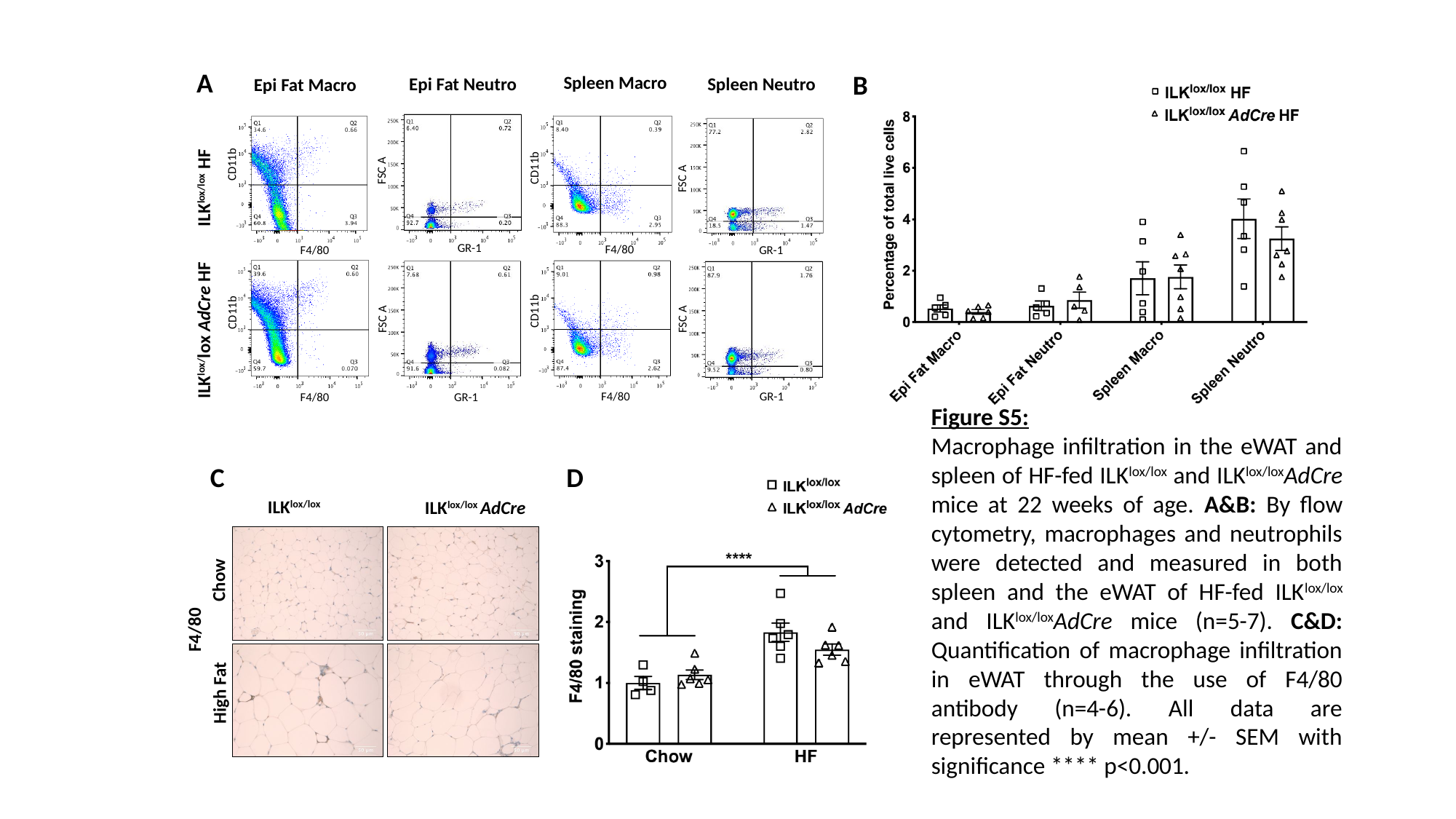

A
B
Spleen Macro
Epi Fat Neutro
Spleen Neutro
Epi Fat Macro
FSC A
CD11b
CD11b
FSC A
GR-1
F4/80
F4/80
GR-1
CD11b
FSC A
FSC A
CD11b
F4/80
GR-1
F4/80
GR-1
ILKlox/lox HF
ILKlox/lox AdCre HF
Figure S5:
Macrophage infiltration in the eWAT and spleen of HF-fed ILKlox/lox and ILKlox/loxAdCre mice at 22 weeks of age. A&B: By flow cytometry, macrophages and neutrophils were detected and measured in both spleen and the eWAT of HF-fed ILKlox/lox and ILKlox/loxAdCre mice (n=5-7). C&D: Quantification of macrophage infiltration in eWAT through the use of F4/80 antibody (n=4-6). All data are represented by mean +/- SEM with significance **** p<0.001.
C
D
ILKlox/lox
ILKlox/lox AdCre
Chow
F4/80
High Fat

## Slide 7
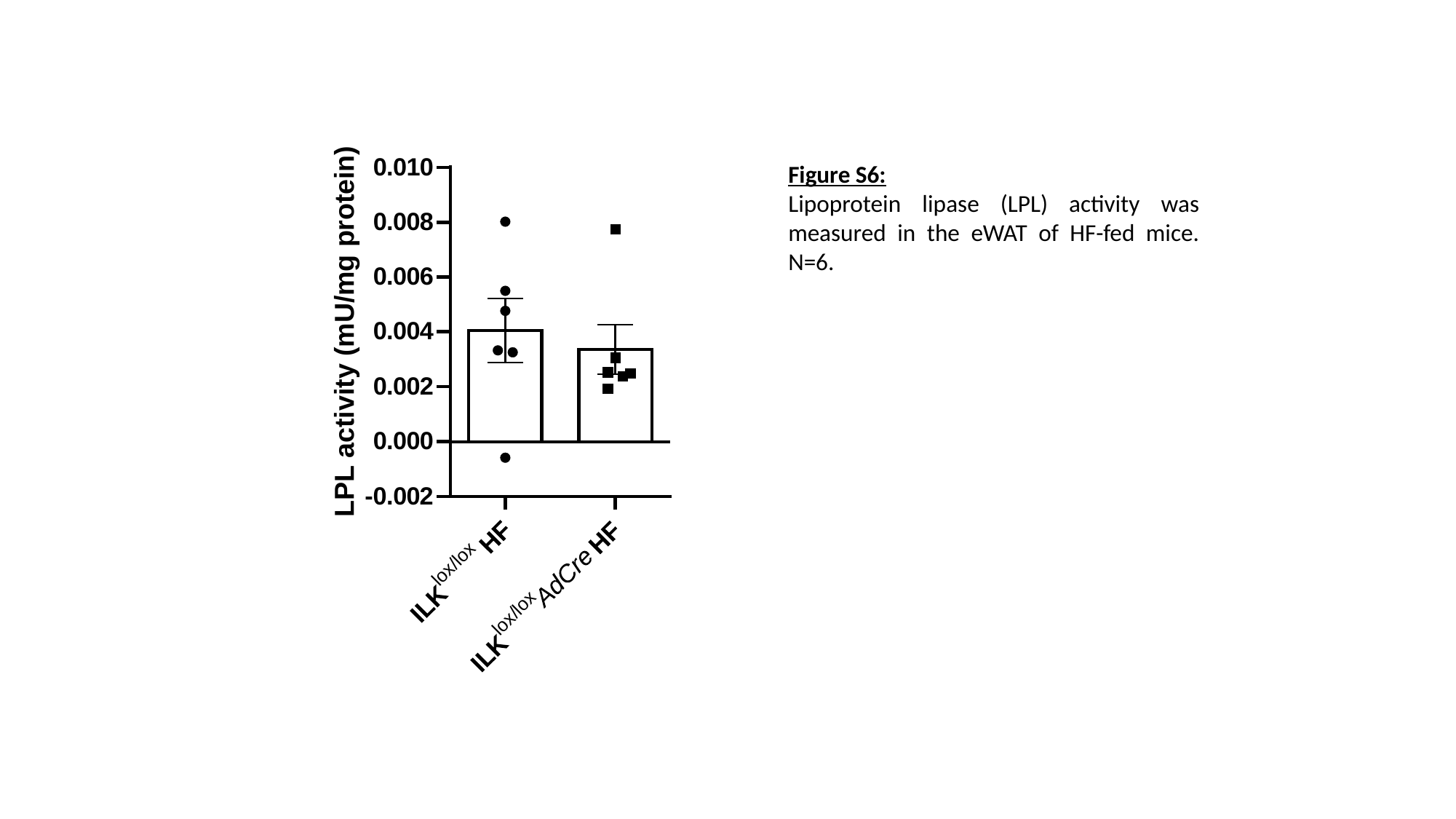

Figure S6:
Lipoprotein lipase (LPL) activity was measured in the eWAT of HF-fed mice. N=6.

## Slide 8
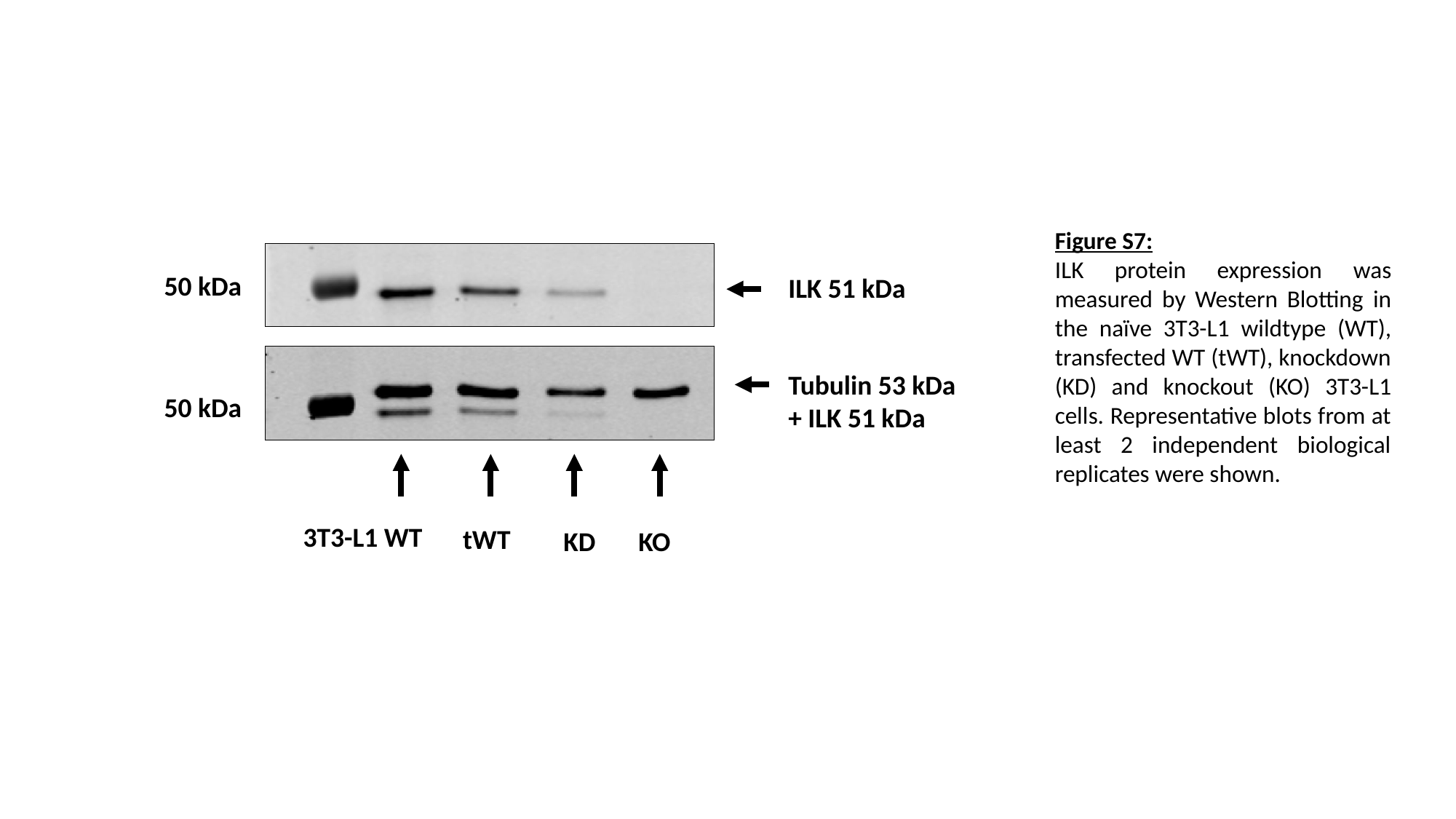

Figure S7:
ILK protein expression was measured by Western Blotting in the naïve 3T3-L1 wildtype (WT), transfected WT (tWT), knockdown (KD) and knockout (KO) 3T3-L1 cells. Representative blots from at least 2 independent biological replicates were shown.
50 kDa
ILK 51 kDa
Tubulin 53 kDa
+ ILK 51 kDa
50 kDa
3T3-L1 WT
tWT
KO
KD

## Slide 9
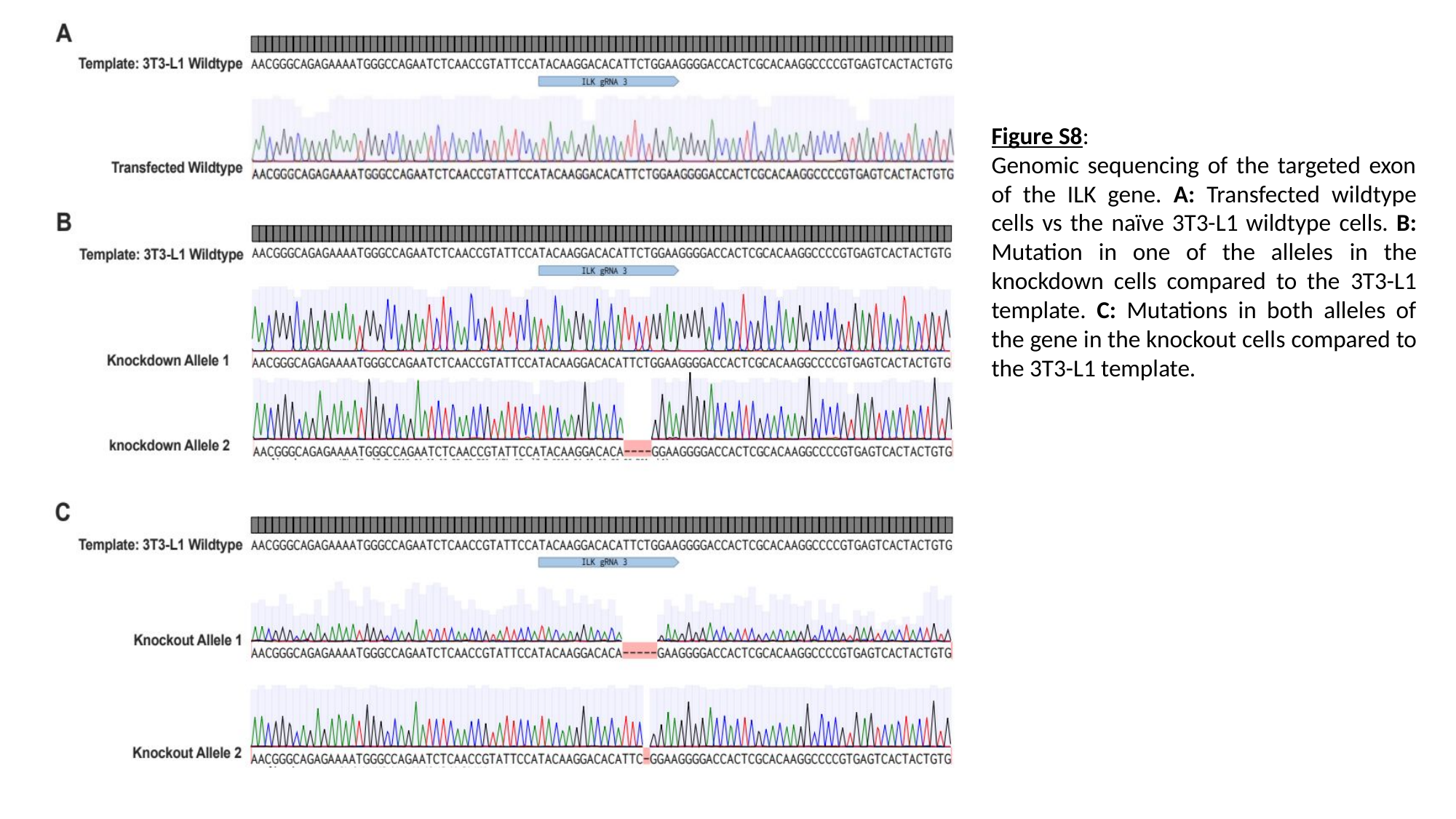

Figure S8:
Genomic sequencing of the targeted exon of the ILK gene. A: Transfected wildtype cells vs the naïve 3T3-L1 wildtype cells. B: Mutation in one of the alleles in the knockdown cells compared to the 3T3-L1 template. C: Mutations in both alleles of the gene in the knockout cells compared to the 3T3-L1 template.
